# Beyond network-based approaches: uncovering the true cost of connectivity

**DOI:** 10.1007/s10980-026-02382-3

**Published:** 2026-05-28

**Authors:** Karolina Argote, Benoît Geslin, Lucyle Hernandez-Possémé, Mailys Queru, Mathieu Santonja, Cécile H. Albert

**Affiliations:** 1 IMBE, Aix Marseille Univ, CNRS, Avignon Univ, IRD, Marseille, France; 2 UMR 6553 ECOBIO, Université de Rennes (UNIR), CNRS, Rennes, France; 3 EPHE, Université PSL, 75005 Paris, France

**Keywords:** Habitat fragmentation, Corridors, Stepping stones, *Folsomia candida*, Landscape context, Connectivity

## Abstract

**Context:**

Fragmented landscapes challenge biodiversity conservation. While connectivity elements are promoted to mitigate the effects of fragmentation, their effectiveness remains debated.

**Objectives:**

This study evaluates the effectiveness of corridors and stepping stones in enhancing dispersal success and population dynamics in a high-resistance matrix. We compare two experimental approaches: a landscape-based design that explicitly accounts for matrix resistance, and a traditional network-based design in which habitat patches are connected by tubes.

**Methods:**

We conducted a laboratory experiment using the microarthropod model *Folsomia candida*, manipulating fragmentation by varying the distance between four habitat patches embedded in a high-resistance matrix. Connectivity elements (corridors and stepping stones) were tested and compared with unconnected landscapes (control) and network-based designs. Over 12 weeks, we monitored colonization success, population size, reproduction rate, and adult survival using AI-based image analysis.

**Results:**

The presence of corridors as a connectivity element enhanced the colonization of initially empty patches, but their effectiveness was distance-dependent. Stepping stones did not differ from the non-connected control landscapes. Interestingly, the largest total populations were found in the non-connected control landscapes, where restricted dispersal resulted in higher reproduction, indicating a trade-off between movement and reproduction. The highest mortality of released adults was observed in landscapes with corridors, whereas higher recruitment was observed in stepping stones landscapes. Network-based designs yielded a higher population size in initially empty patches compared to landscape-scale designs. However, as they bypassed matrix-related dispersal costs, they led to a miss-estimation of the movement, reproduction, survival and recruitment processes.

**Conclusions:**

Connectivity elements can support dispersal and reproduction, but may also increase mortality in resistant matrices. Conservation strategies should incorporate matrix resistance and landscape-scale context to better promote population dynamics in fragmented landscapes.

**Supplementary Information:**

The online version contains supplementary material available at https://doi.org/10.1007/s10980-026-02382-3.

## Introduction

Habitat loss, fragmentation, and degradation are major threats to global biodiversity, contributing to population decline and extinction (Banks-Leite et al. 2020). To address these negative impacts, conservation strategies often focus on enhancing connectivity (i.e., the ability of organisms to move across landscapes) through the implementation of connectivity elements such as corridors and stepping stones (Crooks et al. 2017). Corridors are linear features that connect separate habitat patches directly and continuously, while stepping stones are discontinuous elements that provide intermediate stops during movement, such as small patches of habitat or isolated landscape features (Hilty et al. 2019). These connectivity conservation strategies rely on the assumption that maintaining movement across landscapes promotes dispersal and colonization, both of which are essential for the dynamics and persistence of populations and metapopulations at the landscape scale.

However, although connectivity elements are widely advocated as tools to mitigate fragmentation, their effectiveness is not universal and depends heavily on several key features. These include a) the functional traits of the target species, b) the structural and functional quality of the connectivity elements, and c) the surrounding landscape context. Indeed, while corridors have been shown to enhance movement, genetic diversity, and population persistence (Resasco 2019; Gilbert-Norton et al. 2010), their effectiveness varies depending on species traits, such as dispersal ability, and the spatial and temporal scales considered (Merenlender et al. 2022). In addition, the number and characteristics of connectivity elements, such as their width or the presence of resources, play a critical role in determining their success (Christie & Knowles 2015). The length of the corridor is one of the critical variables, as corridors that are too long may prevent animal dispersal, even if the movement cost is low (Brodie et al. 2025). Regarding stepping stones, despite their potential to facilitate movement across fragmented habitats, their functionality remains controversial, and they should be carefully designed to avoid acting as dispersal traps or exacerbating mortality risks in hostile matrices (Wang et al. 2023; Saura et al. 2014). Finally, the permeability of the surrounding landscape matrix further modulates these dynamics. High-resistance matrices can amplify the need for connectivity elements while also raising the mortality rate of dispersing individuals (Arroyo-Rodríguez et al. 2023; Baum et al. 2004).

Despite a growing body of theoretical and empirical evidence, there are still significant gaps in our understanding of how to design universally effective connectivity elements across taxa and landscapes. Addressing these gaps requires a deeper understanding of the mechanisms through which species interact with connectivity elements in different environmental conditions, as well as robust, context-specific empirical studies to refine conservation strategies. On the one hand, in situ corridor experiments often focus on one or a few corridors in specific landscape contexts, have limited generalizability, and lack the mechanistic understanding needed to fill these gaps (Damschen et al., 2006; Haddad et al., 2003). On the other hand, experiments conducted in miniature landscapes offer a valuable opportunity to better understand the processes at work in fragmented landscapes, while allowing for replication and control of confounding factors (Haddad et al. 2015; Benton et al. 2007). However, to date, most of these miniature landscape experiments have relied on a network-based conceptualization of landscapes, tending to assume simplified connections between habitat patches and omitting the potential effects of the surrounding landscape matrix (e.g., Rayfield et al. 2023; Li et al. 2023; Gilarranz et al. 2017). In these experiments, ecological corridors are often represented by tubes connecting habitat patches, thus potentially acting as channels that guide animal movements. This can lead to inaccurate estimates of key processes such as movement, mortality, and reproduction, ultimately reducing our confidence in what constitutes a landscape favorable to biodiversity (Fletcher et al. 2024; Ramírez-Delgado et al. 2022; Baum et al. 2004).

Here, we address this twofold issue by implementing a new type of mini-landscape experiment, in which habitat patches are embedded within a continuous two-dimensional matrix. Individuals can move freely within this matrix and experience realistic movement costs (Argote et al. 2025). The aim of our experiment is to test the effectiveness of connectivity elements while accounting for the matrix context. We assess the effectiveness of different types of connectivity element (corridors and stepping stones) for a range of inter-patch distances, a potential confounding effect (Brodie et al., 2015). We also compare our landscape-based experimental design to a network-based design, in which habitat patches are connected by tubes. We test and compare both designs using *Folsomia candida*—a microarthropod species that is widely used in connectivity studies—and monitor population size, colonization, reproduction, and survival over 12 weeks (~ 3 generations) (Argote et al. 2023). We hypothesize that:

### H1

Connectivity features (corridors, stepping stones and tubes) enhance colonization (rate and speed) compared to non-connected control landscapes, and these changes are expected to have implications for population size and demographic rates (Bennett & Mulongoy 2006; Ricketts 2001).

### H2

Traditional network-based designs overestimate colonization rates and population sizes compared to landscape-based designs, due to the channeling effect of connecting tubes and reduced movement-related mortality (Fletcher et al. 2011).

## Material and methods

### Material collection

#### Preparation of Collembola cohorts

*Folsomia candida* (Willem 1902) is a well-studied Collembola species due to its parthenogenetic nature and short reproductive cycle, rendering it suitable for laboratory studies (Fountain & Hopkin 2005; Usher & Stoneman 1977). The population used was sourced from rearing of the Mediterranean Institute of Biodiversity and Ecology (IMBE, Argote et al. 2023). Individuals were reared in vials (5.5 cm diameter × 9.5 cm height) with a 9:1 plaster of Paris–charcoal substrate at 20 ± 2 °C. To synchronize cohorts, adults were kept at 4 °C for 2 days to induce egg-laying, and eggs were transferred to clean vials (Biryol et al. 2024). After 3–4 days, newborns were fed dry yeast pellets until 22–26 days old.

### Leaf litter collection

Senescent leaves were collected from *Quercus pubescens* trees during November–December 2019 on the Lure Mountain, located in the Luberon Natural Regional Park in south-eastern France. Prior to laboratory experiment, to allow the leaching of secondary metabolites potentially toxic to Collembola and the colonization of microorganisms (Biryol et al. 2024; Aupic-Samain et al. 2021), leaves were placed in litterbags on the soil surface for 40 days at the Oak Observatory of Haute-Provence (O3HP experimental site, 43°56′6.72″ N 5°42′38.52″ E), near Lure Mountain. After collection, samples were air-dried for 24 h, frozen twice at − 18 °C for 48 h to remove fauna, and stored in darkness at ambient temperature until the start of experiment (Aupic-Samain et al. 2019; Santonja et al. 2018).

## Experimental setup

**Fig. 1:**
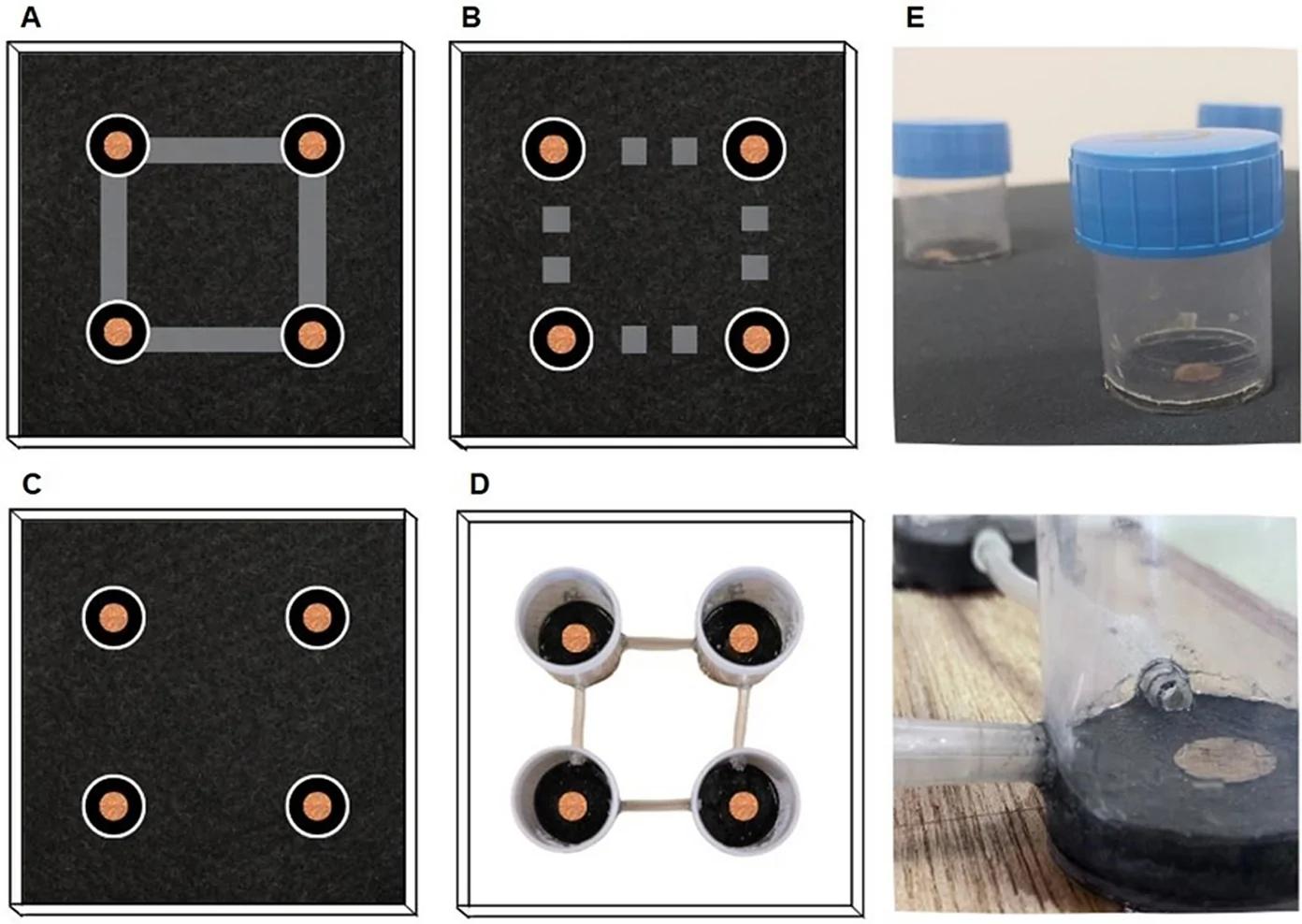
Experimental design with four landscape types: landscape-based (**A**, **B**, **C**) and network-based (**D**). Each had four habitat patches with a *Quercus pubescens* disc for shelter and resources. In the landscape-based design, habitat patches were embedded in a high-resistance matrix (felt) and connected by: **A**) low-resistance corridors (cotton strips), **B**) low-resistance stepping stones (cotton pieces), or **C**) no connectivity elements. In the network-based design (**D**) habitat patches were connected via plastic tubes. **E** Zoomed view of habitat patch vials and openings in both designs: horizontal slots (landscape-based design) or connected tubes (network-based design)

The experiment included 48 artificial landscapes with a fixed habitat amount (i.e., 4 habitat patches, representing 2% of the landscape) and an 8-level inter-patch distance gradient (5, 8, 11, 15, 20, 25, 30, 38 cm), with or without connectivity elements (Fig. 1). These distances can be easily traversed by F. candida in a matter of minutes when the landscape matrix is permeable (a mixture of plaster and charcoal; Argote et al. 2023), or –for longer distances– remain impassable for weeks when the matrix is highly resistant (Argote et al. 2025). Habitat patches were cylindrical vials (3.4 cm diameter × 7 cm height) half-filled with a 9:1 plaster of Paris–charcoal substrate, identical to *F. candida* rearing conditions. Each patch contained a 1-cm *Q. pubescens* litter disc, providing food, shelter, and multi–generational population support (Argote et al. 2023). *Folsomia candida* typically requires moist conditions to survive and reproduce, as the ones offered by the rearing substrate we use as habitat. The range of inter-patch distances selected is within the reach of *F. candida* individuals when moving on a permeable substrate (23 cm max. in 10 min, Argote et al. 2023). Therefore, observations of limited colonization of initially empty patches can be attributed to matrix resistance and connectivity elements, rather than movement capacity. Starting with 20 individuals, habitat patches were large enough to host population of up to 240 individuals after 6 weeks (median = 180 individuals, Argote et al. 2025). Therefore, observations of small populations can be attributed to spatial effects (movement-related mortality) and not to patch carrying capacity. Materials serving as landscape matrix between the habitat patches were selected based on prior experiments assessing *F. candida* colonization success across different materials (SI-1). Argote et al. (2025) showed that felt corresponds to a high-resistance matrix where it is harsher for individuals to move and on which they quickly die, leading to low levels of colonization. Contrastingly, organic cotton fabric was shown to be a low-resistance matrix leading to less quick death and higher colonization rates for wandering individuals. Cotton was glued with textile adhesive (Prym®), sterilized with ethanol, frozen (-2 °C, 48 h), and subjected to thermal shock (200 °C) to remove contaminants.

### Landscapes design types

The experiment used two types of design (Fig. 1).

#### Landscape-based design

50 × 50 × 10 cm plastic boxes were filled with a high-resistance felt matrix, with habitat patches including horizontal slots to allow *F. candida* movement. This design included three landscape types. Non-connected control landscapes lacked connectivity elements (N = 8; once per inter-patch distance). In contrast, in landscapes with corridors (N = 16; each inter-patch distance repeated twice), four strips of organic cotton, each 1.5 cm-wide, connected habitat patches. In landscapes containing stepping stones (N = 16; each inter-patch distance repeated twice), 1 × 1 cm organic cotton squares spaced 1 cm apart, formed intermittent connections between patches.

#### Network-based design

This design included four habitat patch vials connected by flexible plastic tubes (4.8-mm outside diameter, 0.8-mm wall thickness) following Gilarranz et al., (2017); each vial was perforated twice to connect the tubes. This treatment was not replicated (N = 8).

In both design types, 20 individuals (22–26 days old) from the same cohort were placed in one of four habitat patches in each landscape or network. The experiment lasted 12 weeks (~ 3 generations) and was conducted in two blocks. Landscapes were maintained at 20 ± 2 °C in a dark room. Weekly, vials were aerated, hydrated with 2 ml distilled water, and photographed to monitor and count individuals using YOLOv5, an AI-based tool trained to detect *F. candida* (SI-1).

### Response variables

We calculated eight complementary response variables:

Regarding population dynamics, we estimated each week (1) the total population size at the landscape scale and (2) the total population size in target (initially empty) patches. Regarding the reproduction of released individuals, we assessed (3) the number of juveniles over time in the landscapes and (4) the maximum size reached by the population.

Regarding the **survival and growth of individuals**, the number of adults in the population reflects both the survival of released individuals and the recruitment of juveniles that have reached adulthood. The (5) number of adults in the first few weeks (before individuals born in the landscape could reach adult size, i.e., weeks 1 to 3) reflects the mortality of released individuals, while (6) the number of adults in subsequent weeks (weeks 8 to 10) represents a balance between the two, with recruitment potentially outweighing mortality (weeks 8 to 10; (6)). The three-week windows correspond to approximately one generation for *F. candida*. Regarding **colonization success**, we assessed (7) the number of colonized patches each week and (8) the date of first colonization in each landscape.

It should be noted that we differentiated juveniles born in the landscapes of released adults based on their body length, estimated using the YOLO-Collembola tool (SI-1). During the first week, only adults were present in the landscapes, with juveniles emerging only in the subsequent weeks. The histograms of body length estimates over the weeks are clearly bimodal (SI-1). We therefore assumed that typical healthy breeding adults, such as those released that had been raised under ideal laboratory conditions (moist substrate, 20 °C, unlimited food, ~ 21 days), can be distinguished from juveniles or sub-adults with a length threshold of 0.85 mm (SI-1).

## Statistical analysis

Different model types were required to accommodate the varied data structure and distributions of the response variables.

The total population size at landscape (1) and patch scale (2), the number of juveniles (3), the number of adults (weeks 1–3 (5) and 8–10; (6)) and the number of colonized patches (7), were modelled using Generalized Linear Mixed Models (GLMMs, package *glmmTMB*). To account for temporal autocorrelation, models included a first-order autoregressive (AR1) variance structure (Wenger et al. 2022). To account for the fact that the different designs and repetitions were ran in temporal blocks, models included a random block effect. With this random structure, we compared different error distributions (Poisson, Negative binomial type 1 and 2) and zero-inflation structures (~ 1 or ~ inter-patch distance) to accommodate count and zero-inflated data and lead to satisfying residuals distributions (normal distribution, uniformity, over-dispersion). Finally, we tested all possible combinations of fixed explanatory variables: inter-patch distance (log-transformed, linear and quadratic terms tested), connectivity type (factor: none, network, corridors, stepping stones), and their interaction. Models with different fixed effects were compared by their Akaike Information Criterion (AIC) and we kept the best model on the basis of the smallest AIC; ΔAIC > 2 (SI-2).

We investigated the maximum population size (4) using a generalized linear model (GLM) with a normal error distribution. Explanatory variables included the inter-patch distance, the connectivity type and their interaction. Resulting models were compared by their AIC (SI-2). Regarding colonization, we investigated the time of first colonization (8) using a Cox proportional hazards model which evaluates the effect of several factors on the rate of a particular event happening in time (i.e. death, Cox 1972). Models with all combinations of inter-patch distance (log-transformed and linear) were also performed and compared by their AIC (SI-2).

We then performed post-hoc multiple comparisons with Tukey adjustment to compare responses between connectivity treatments at 5 cm, 19 cm and 38 cm and between distances within connectivity treatments when relevant.

All statistical analyses were conducted in R 4.2.3 (R Core Team 2023), using the following packages: stats, lme4 (ver. 1.1–34; Bates et al. 2015), glmmTMB (ver. 1.1.6; Brooks et al. 2017); DHARMa (ver. 0.4.6; Hartig 2022); emmeans (ver. 1.8.5; Lenth 2023), survival (ver. 3.5–5; Therneau & Grambsch 2000), performance (ver. 0.10.4; Lüdecke et al. 2021), survminer (ver. 0.4.9; Kassambara et al. 2021), ggResidpanel (ver. 0.3.0; Goode & Rey 2019) for statistical analyses, and sjPlot (ver. 2.8.13; Lüdecke 2023), ggplot2 (ver. 3.4.1; Wickham 2016) and RGraphics for generating graphs.

## Results

### Population size

**Fig. 2:**
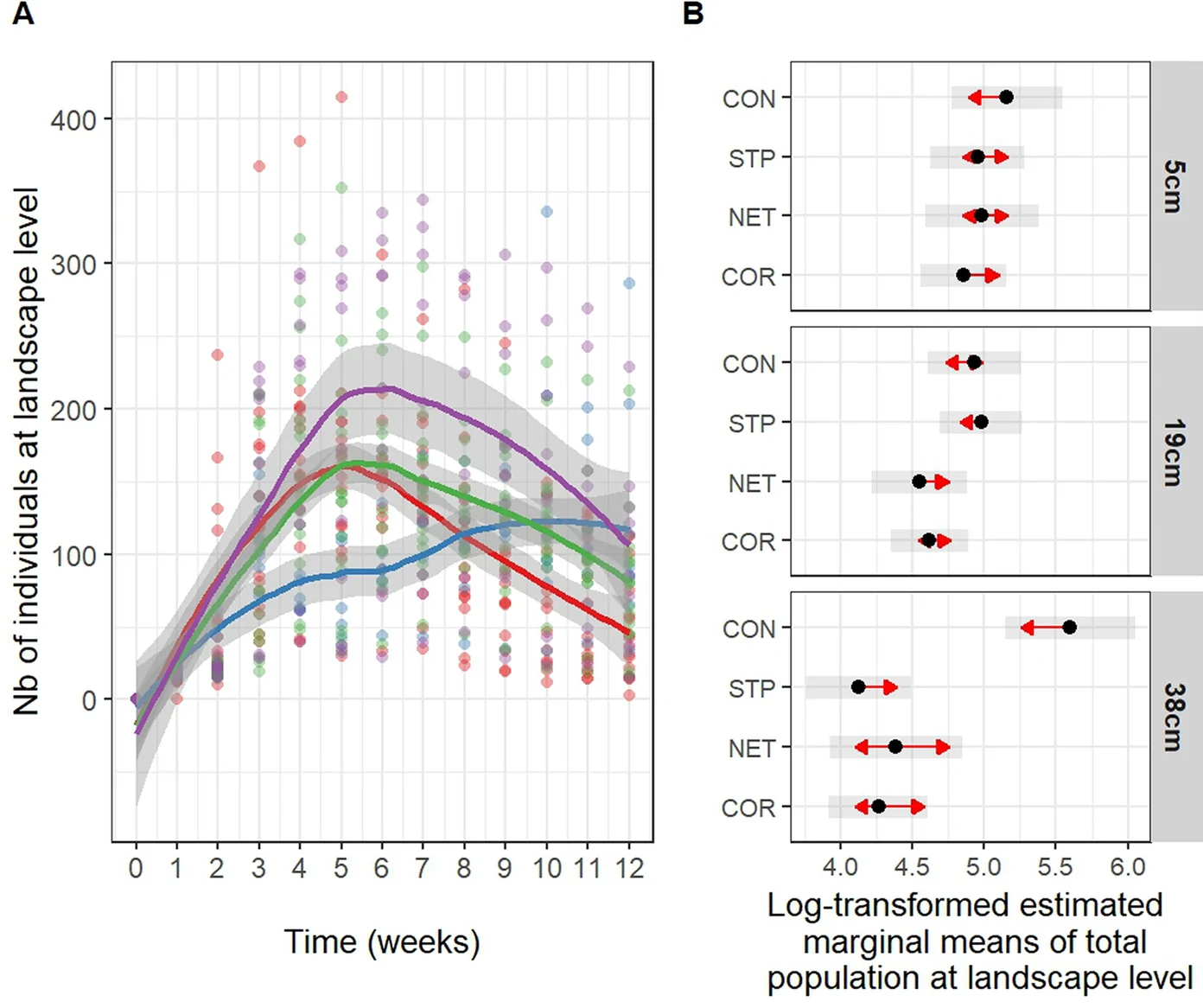
Total population size at landscape level by connectivity treatment: **A** Number of individuals in landscapes over time. Points represent all landscapes in time. Line colors represent treatments (loess smoothing): non-connected control landscapes (purple), stepping stone (green), network (blue), and corridor (red). Grey envelopes display 0.95 confidence intervals. **B** Estimated marginal effects of connectivity type at minimum, median and maximum distances. The grey bars are confidence intervals. The red arrows are for the comparisons among them, overlapping arrows display non-significant pairwise differences. COR = corridors, NET = networks, STP = stepping stones, CON = non-connected control.

**Fig. 3:**
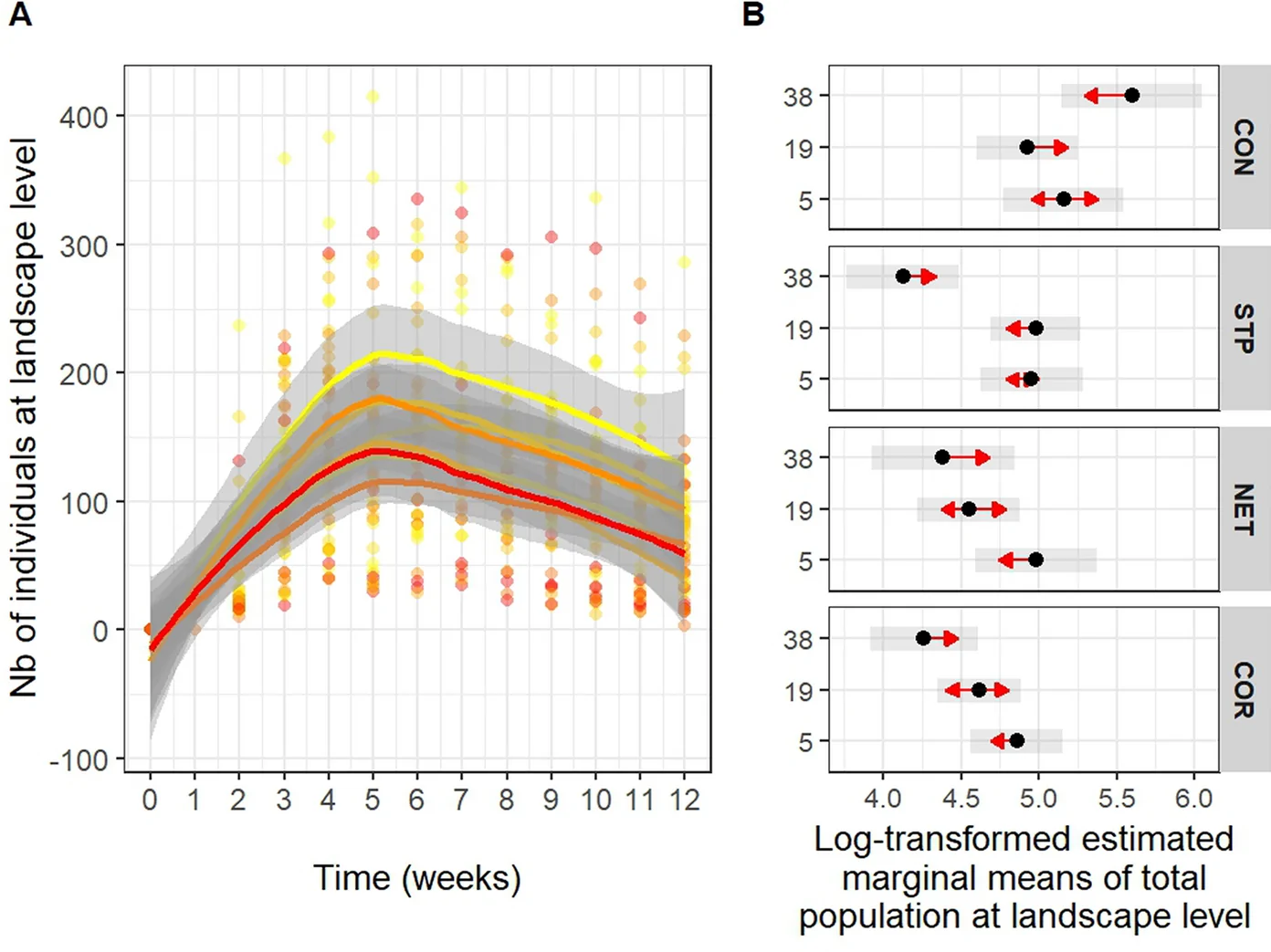
Total population size at landscape level by inter-patch distance: **A** Number of individuals in landscapes over time. Points represent all landscapes in time. Line colors represent inter-patch distances (loess smoothing): from short distances in yellow, to large ones in red. Grey envelopes display 0.95 confidence intervals. **B** Estimated marginal effects of inter-patch distance for each connectivity treatment. The grey bars are confidence intervals. The red arrows are for the comparisons among them, overlapping arrows display non-significant pairwise differences. COR = corridors, NET = networks, STP = stepping stones, CON = non-connected control

The best model to explain total population size at the landscape scale over 12 weeks included significant effects of inter-patch distance (linear and quadratic terms), connectivity type, and their interaction (lowest AIC, ∆AIC with additive model = 22; Fig. 2; SI-2). At the shortest inter-patch distance (5 cm), no significant difference in population size was observed neither among the connectivity treatments (corridor, network and stepping stone) nor with the non-connected control treatment. At the intermediate distance (19 cm), population size was significantly higher in stepping stone than in corridor (p-value = 0.007) and in network landscapes (p-value = 0.008); and tended to be larger in control than in networks (p-value = 0.07, SI-2). At the longest distance (38 cm), the Non connected control landscape had larger population size than network, corridor, and stepping stone landscapes (p-values = < 0.0001, SI-2). No significant difference was detected among the connectivity treatments at this distance. Non connected control landscapes hence showed higher population size at intermediate and long inter-patch distances, likely because individuals remained in the original patch and reproduced instead of dispersing. Overall, populations also tended to be smaller at larger inter-patch distances for all connectivity treatments, but to be larger at the largest inter-patch distance in the control landscapes (Fig. 3; SI-2).

The best model to explain the population size in target patches (i.e., initially empty patches) included significant effects of inter-patch distance (linear and quadratic terms), connectivity type, and their interaction (lowest AIC, ∆AIC with additive model = 5; SI-2). At 5 cm, the non-connected control landscape had significantly lower population size than the others (all p-values < 0.05) and the network landscapes tend to have larger population size than the others (all p-values < 0.1, SI-2). No significant difference was found between the corridor and stepping stone landscapes. At both the intermediate (19 cm) and longest (38 cm) distances, no significant difference was observed across the different landscape types. Hence, significant difference in population size in target patches was only found at the shortest distance (5 cm), where networks tend to exhibit larger population size than corridor and stepping stone landscapes and all had larger population size than the control landscape. The number of individuals in target patches was also significantly larger in landscapes with short inter-patch distances, for all the connectivity types (SI-2).

## Reproduction

During the first few weeks, the released adults reproduced in the landscapes, leading to a sharp increase in population size (from 20 to several hundred individuals) and meaning that the populations contained a large proportion of juveniles (all individuals smaller than the released individuals). For this reason, the temporal evolution of the number of juveniles was largely equivalent to that of the total population size, resulting in highly similar models (SI-2). The temporal evolution of population size was fairly similar between non-connected control, stepping stone and corridor landscapes, with a sharp increase in the first few weeks, a peaked reached around week 5–6, and then a decline. However, the evolution of networks was different, with a slower increase that continued throughout the experiment (Fig. 2). Reproduction peak was particularly strong in control landscapes and at large distances (Fig. 2 and 3). Yet, maximum population size did not differ significantly among connectivity types, but it decreased with increasing inter-patch distance, meaning reproduction was greater at shorter inter-patch distances (Fig. 3; SI-2).

## Survival and growth

**Fig. 4:**
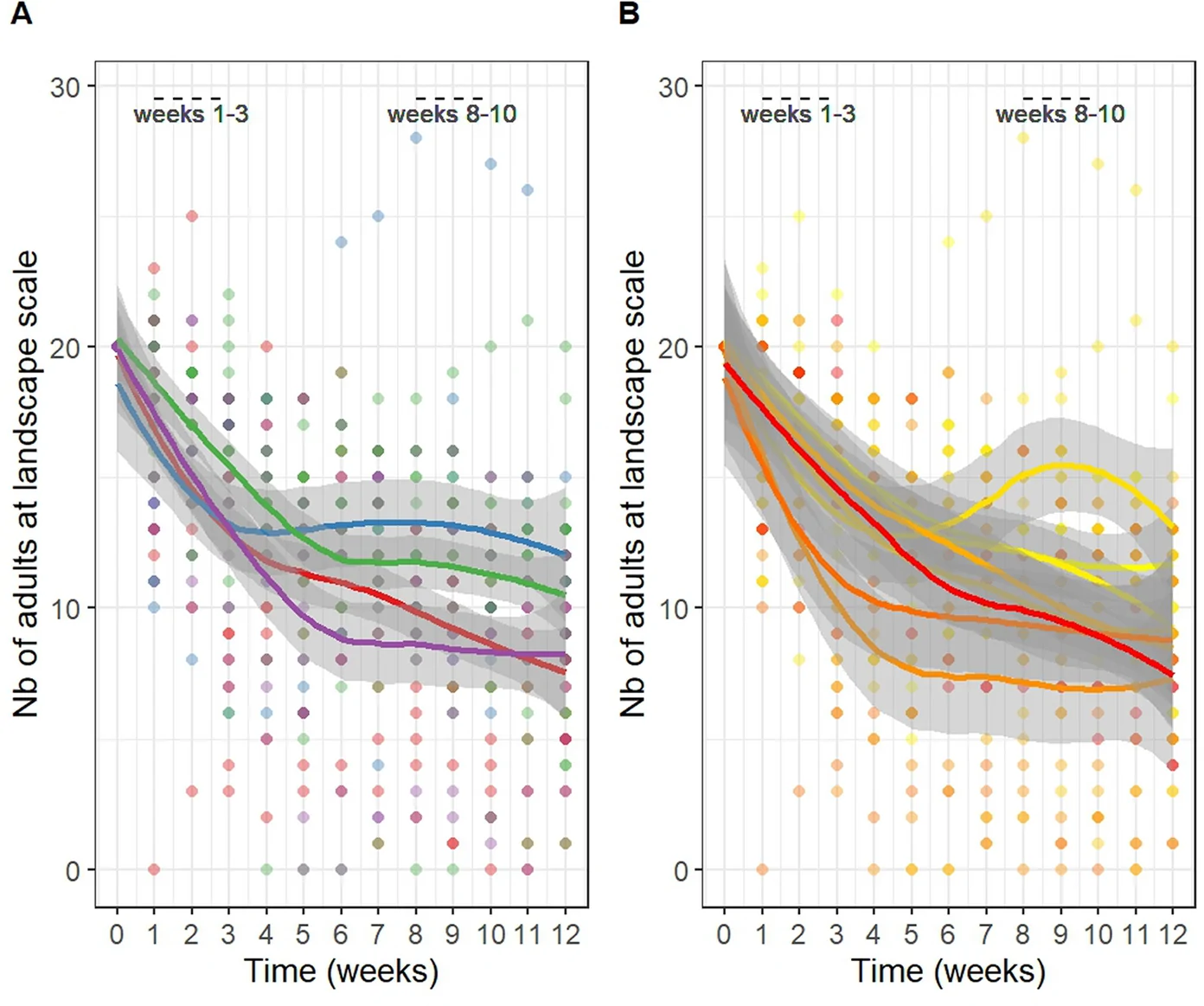
Temporal dynamics of the number of adult-size individuals: **A** Points represent all landscapes in time. Lines represent average temporal trend for connectivity treatments (loess smoothing): non-connected control landscapes (purple), stepping stone (green), network (blue), and corridor (red). **B** Lines represent average temporal trend by inter-patch distance (loess smoothing): from short distances in yellow, to large ones in red. Grey envelopes reflect 0.95 confidence intervals. The two periods analyzed (weeks 1–3 and weeks 8–18) are displayed

**Fig. 5:**
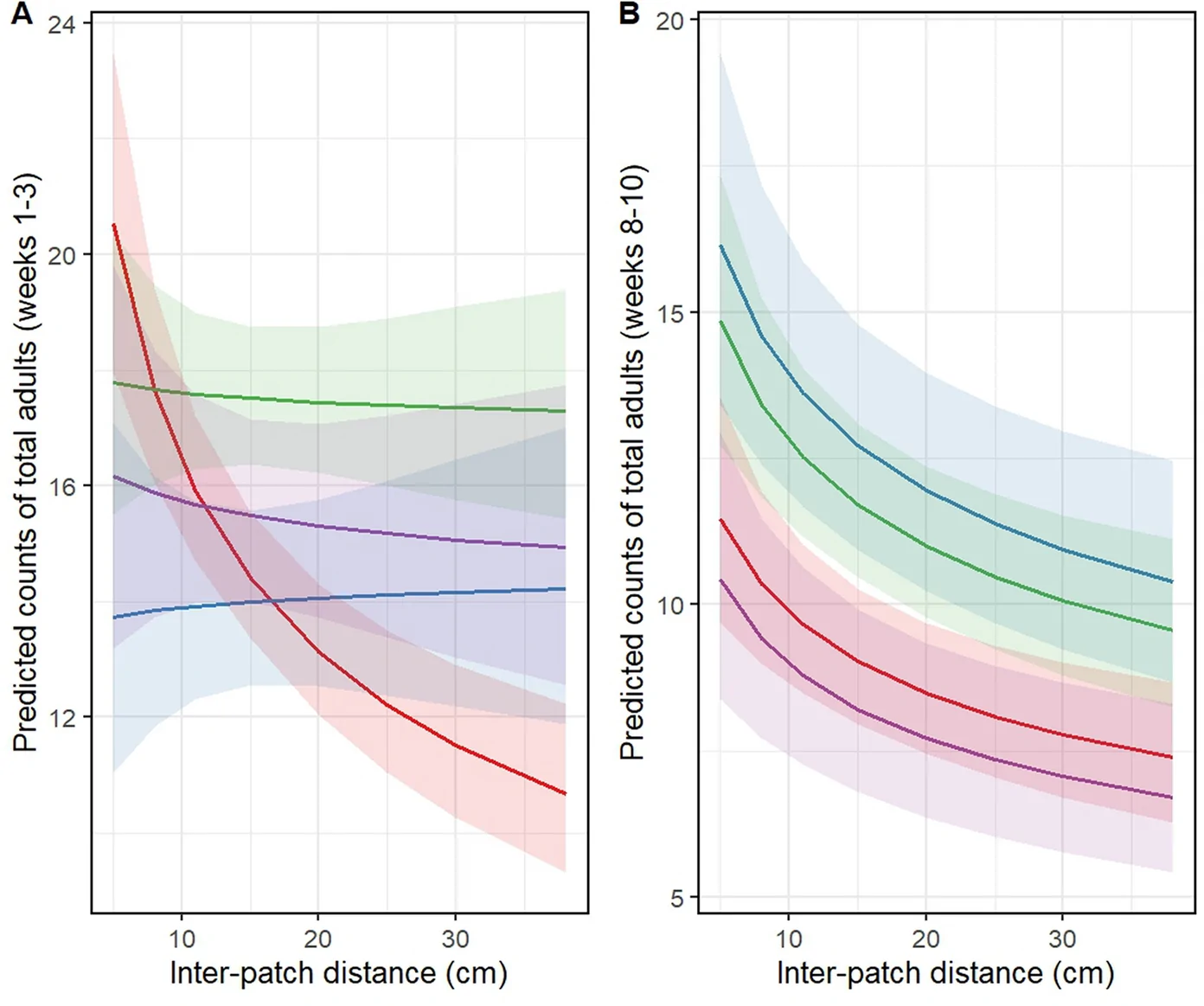
Predicted effect of inter-patch distance and connectivity type on the number of adults in landscapes based on the best-fitted models: **A** Dynamics over weeks 1 to 3; **B** Dynamics over weeks 8 to 10. Lines represent different types of connectivity elements: non-connected control (purple), stepping stones (green), networks (blue) and corridors (red). Shaded areas display the associated 95% confidence intervals

The number of large individuals, identified as healthy adults, decreased during the first three weeks of the experiment in all types of landscape (Fig. 4). As juveniles born in the landscapes were still very small during this period (SI-2), this suggests that the released adults died in the meantime. The best model to explain this dynamic includes the effects of inter-patch distance (linear or log-transformed terms lead to equivalent models), landscape type and their interaction (lowest AIC, ∆AIC with additive model = 15; SI-2). While the number of adults remained fairly stable with increasing inter-patch distance for networks, non-connected control and stepping stone landscapes, it decreased sharply for corridor landscapes (Fig. 5). This means that corridors associated with large inter-patch distances increase the mortality of released individuals. Overall, the mortality in networks tended to be lower than in corridor landscapes at larger distances (p = 0.06) and was higher at short distances (p = 0.01, SI-2).

The number of large individuals, identified as healthy adults, remained fairly stable between weeks 8 to 10, peaking in some landscapes and exceeding the number of individuals released (> 20), despite mortality. This suggests that juveniles born in these landscapes grow large enough to become healthy adults. The best model to explain this dynamic includes the effects of inter-patch distance (linear or log-transformed terms lead to equivalent models) and landscape type without interaction (lowest AIC, ∆AIC with model with interaction model = 4; SI-2). The number of adults recruited decreased with increasing inter-patch distance (Fig. 5). Recruitment was also higher in stepping stone and network than in corridor and control landscapes (all p-values < 0.01, SI-2).

## Colonization success

The best model explaining the number of colonized patches over the 12-week experiment included significant effects of inter-patch distance (log-transformed), connectivity type and their interaction (lowest AIC, ∆AIC with additive model = 17; Fig. 2; SI-2). The number of patches colonized consistently decreased with increasing inter-patch distance across all treatments with a steep decline between the smaller and intermediate distances. Fewer patches colonized also means the population remains more spatially aggregated in one or a few patches. At short inter-patch distances (5 cm), the number of colonized patches did not differ among colonization treatments. At intermediate inter-patch distances (19 cm), corridors significantly outperformed stepping stone and non-connected control treatments (all p-value < 0.005, SI-2) while no significant differences were observed among networks, stepping stones, and control. At the longest inter-patch distance (38 cm), corridors led to higher levels of colonization than the other treatments (all p-value < 0.1, SI-2); while the other treatments were not significantly different.

Colonization speed differed among landscapes, with the best model including the effects of inter-patch distance, connectivity type and their interaction (equivalent to additive model and close to model with inter-patch distance only, SI-2). Hence, colonization is significantly slower as inter-patch distance increases (estimate -0.04 ± 0.005, z-test = -7.7, p-value = 9.36e-15). Regarding the interaction, colonization is also quicker in corridor than in stepping stone landscapes at medium and large distances, all other contrasts being non-significant (SI-2).

## Discussion

Our connectivity treatments (type of connectivity elements and type of design) led to contrasting demographic and movement outputs, which also depended on distance between habitat patches. These results show that it is essential to understand the demographic and movement processes underlying the effects of connectivity elements in order to accurately assess their effectiveness. They also suggest that a set of complementary indicators are needed to properly assess the effectiveness of connectivity elements. For instance, while the number of individuals at the landscape scale was the highest the control treatment, i.e. in the non-connected landscapes, it did not reflect colonization dynamics and patch occupancy, key factors for metapopulation stability. Below, we discuss the three main elements of these results: 1) the role of inter-patch distance on connectivity, 2) the differences between connectivity elements in how they alter demographic dynamics, and 3) the overall discrepancies between network- and landscape-based designs.

### Inter-patch distance shapes the effectiveness of connectivity elements

Our results confirm that corridors enhance the inter-patch movements observed here as the colonization of initially empty patches (Gilbert-Norton et al. 2010), and lead to a broader occupancy of the landscape compared to the non-connected control treatment. This is only true at large and intermediate distances, as all treatments were equivalent at small distances. This is consistent with previous studies emphasizing that hostile matrices impose severe dispersal barriers to organisms (Haddad et al. 2017). Previous studies have also shown that corridors implemented in such harsh matrices may lose their effectiveness over longer distances (Hodgson et al. 2011; Baum et al. 2004). Here, corridors are still effective to enhance colonization, even though the number of patches colonized declines with increasing inter-patch distance. However, we also note that this goes with a strong increase in the mortality of released adults (Argote et al. 2025); individuals take higher risks in the landscapes with corridors and long inter-patch distances, which is not observed in the other landscapes. If inter-patch distance has long been identified as a potential confounding effect when investigating the effectiveness of corridors (Gilbert-Norton et al. 2010), it is compulsory to control for it (Resasco 2019), as we did here for a good understanding of processes. Our results provide no evidence to support the effectiveness of stepping stones in enhancing colonization, contrary to previous studies (Saura et al. 2014; Wang et al. 2023). This may be due to the harshness of the matrix (Baum et al. 2004), as it is expected that less harsh matrix permeability would encourage attempts of crossing (Donald & Evans 2006), which is required many times in the case of stepping stones.

In our experiment, the felt matrix was chosen as a harsh matrix (10% survival when released at 6.5 cm from a habitat patch on this matrix, SI-1), and corridors and stepping stones did not provide additional resources to *F. candida* (all individuals died within < 1 h when wandering on cotton fabric). Hence, the corridors here did not function as additional habitat but only as movement providers. This contrasts with many corridor studies in which corridors may be seen as degraded habitats in which organisms can find some resources or shelter even if not sufficient to complete their entire life-cycle (Christie & Knowles 2015; Haddad et al. 2015). This may explain the strong inter-patch distance effects we observe and especially the high mortality associated with the very long corridors. This also means that, even though corridor length was increased, the total amount of available habitat remained constant across landscapes. This is a necessary condition for disentangling the effects of habitat amount from those of habitat fragmentation on biodiversity (Fahrig 2019; Fahrig 2026). Similarly to Argote et al. (2025), here the amount of habitat at the landscape scale and the fragmentation level (number of patches) are held constant, but the amount of habitat reachable (Villard & Metzger 2014) by the organisms given the connectivity elements they can encounter (related to spatial isolation) differs greatly among treatments, leading to contrasting demographic outcomes.

### Connectivity elements alter demographic dynamics

At the landscape scale, populations were larger in the non-connected control landscapes, which was not what we expected. This outcome may be due to three main concurrent factors:

First, individuals died faster in the non-connected control treatment than in the landscape with corridors, but the trend is reversed at larger inter-patch distances (Fig. 5). This could be due to the fact that in landscapes with corridors, individuals are more likely to leave their release patch because they first encounter a low-resistance matrix (Argote et al. 2025). Furthermore, they are more exposed to death when exploring longer distances. In contrast, in the non-connected control landscape, individuals always encounter a high-resistance matrix and may be less likely to leave their release patches (flat mortality with increasing distance, Fig. 5). Likelihood that individuals leave their release patch may be intermediate with stepping stones because after encountering a low-resistance matrix, they will periodically encounter the high-resistance one. The likelihood of individuals to leave the habitat patches is unlikely to be purely density-dependence related, as carrying capacity of these experimental patches is not reached in this experiment (90% of patch/week presented population of less than 100 individuals, whereas populations in these patches can reach over 200 individuals, Argote et al. 2025).

Second, the highest reproductive rates were observed in the release patches of the non-connected control landscapes, especially those with high-inter-patch distances, i.e., those landscapes with the lowest colonization rates. This suggests that a trade-off may exist between the allocation of energy to movement and reproduction (Ziv & Davidowitz 2019). Individuals trapped in highly isolated patches may allocate more energy to reproduction while remaining in safer zones (Valente et al. 2023).

**Fig. 6:**
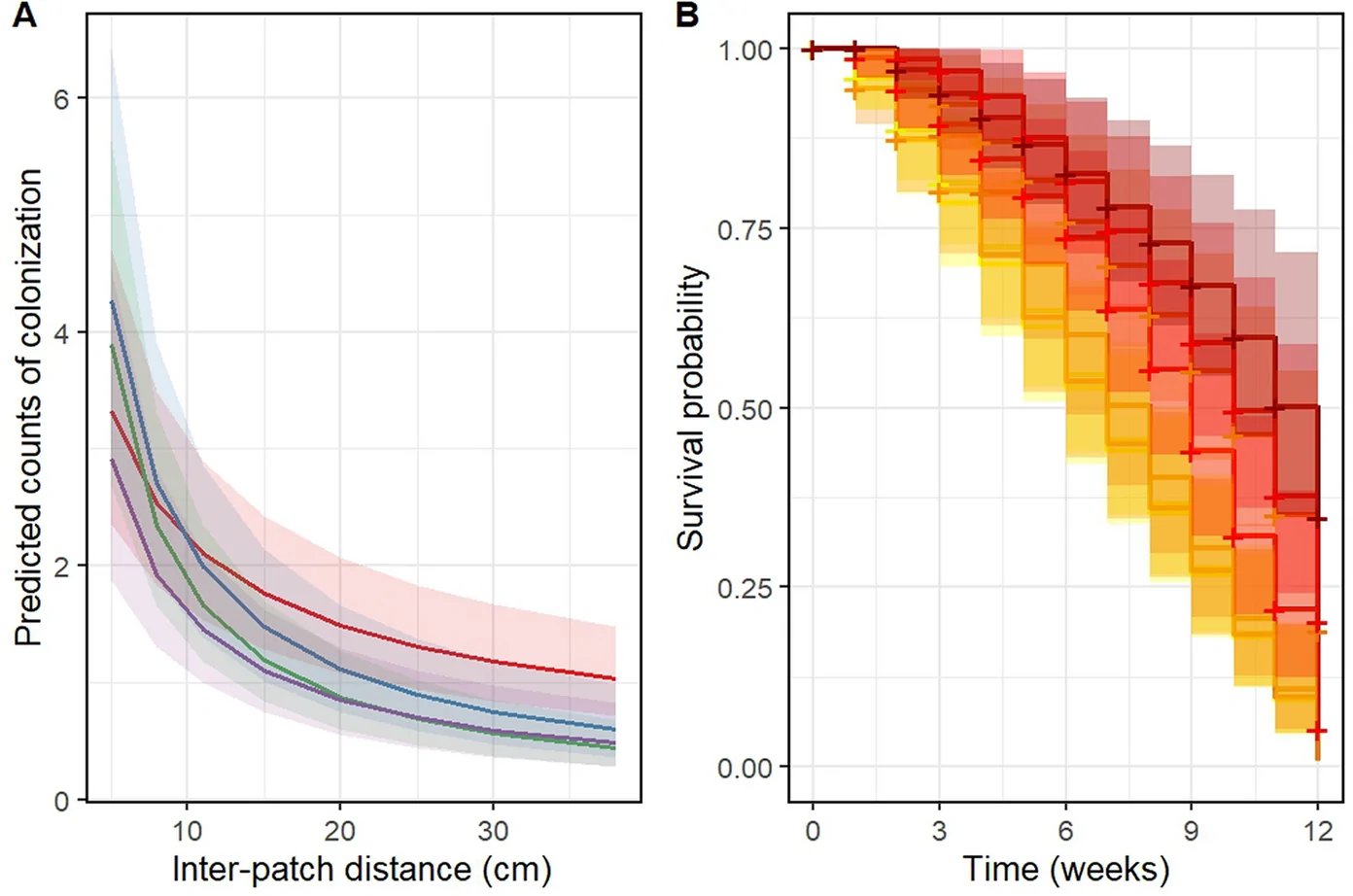
Predicted effect of inter-patch distance and connectivity type on colonization: **A** best-fitted model for the number of colonized patches, based on the. Lines represent different types of connectivity elements: non-connected control (purple), stepping stones (green), networks (blue) and corridors (red). Shaded areas display the associated 95% confidence intervals. **B** Representation of Cox proportional hazards (coxph) model for time of first colonization as an event. “Survival probability” means probability that no colonization occurred. Color from yellow to red represent the gradient of inter-patch distances from small to large

Third, the short duration of our experiment (approximatively 3 generations of *F. candida*) may not fully capture the long-term population viability. High reproduction rates in the release patches in the non-connected control landscapes do not guarantee that populations will not decline due to isolation within one or a few more generations, particularly given the high mortality rate in the matrix (e.g. Yamaura et al. 2022; Day et al. 2020). High mortality, combined with a high propensity for entering the matrix can create dispersal traps (Vasudev et al. 2015; Fletcher et al. 2014). In addition, populations in landscapes with high population sizes and high natality rates, are concentrated in the single release habitat patch. This makes them highly vulnerable to any potential disturbances and increases their risk of extinction (Eriksson et al. 2014). Finally, populations in non-connected control landscapes are also associated with low recruitment rates meaning few juveniles are reaching the size of healthy adults (Fig. 5); which may again exacerbate sensitivity of these populations to disturbances or even to extinction (Fig. 6).

Therefore, it is crucial to understand how demographic parameters are affected by different connectivity treatments in order to predict their ultimate impact on population size and risk of extinction. Here, we show that all the processes related to population dynamics, including reproduction, recruitment, and survival are affected by connectivity types and their interaction with inter-patch distance. Even if none of the population went extinct during the course of our experiment, this has been observed in previous, similar experiments (Argote et al. 2025) which also suggests that we may have observed it with a longer experiment (declining population by week 12). Populations in landscapes with short inter-patch distances appear to be the ones the least at risk, as they exhibit low mortality rates, intermediate natality rates, higher recruitment rates and higher dispersal success (Argote et al. 2025).

### Network-based designs overestimate the benefits of connectivity

When comparing network-based with landscape-based designs, we found that networks led to different birth (mainly lower), recruitment (mainly larger) and mortality (mainly lower) rates than corridor and stepping stone landscapes, and different interaction of inter-patch distance with these effects. These observations could be due to the fact that wandering individuals have a reduced risk of getting lost and dying, which could also lead to reduced energy expanses and therefore more energy allocated to the reproduction (Ziv & Davidowitz 2019). This might be due to the channeling effect of the tubes, which guide individuals from one habitat patch to another. Once individuals enter a tube, they have only two options: move forward towards the habitat or return to their release patch. This reduces the risk of getting lost, and while some individuals may die in the tubes, this happened relatively infrequently. Network-based design also led to increased colonization over non-connected control landscapes over short distances, in a manner similar to corridor and stepping stone landscapes. However, this effect disappeared at larger distances, at which point corridor landscapes were more efficient. In our case, reduced colonization also corresponds to fewer colonized patches, meaning populations are more spatially aggregated in network-based treatments than in treatments with corridors. Finally, the temporal dynamics of populations in network-based treatments differed from those observed in landscape-based ones. Due to reduced movement-related mortality, populations within networks experienced a slower decline in abundance over time, potentially making them less susceptible to extinction. At short inter-patch distances, population sizes were similar in network- and landscape-based treatments. However, as the distance between patches increased, the network-based treatments led to larger and more aggregated populations than the landscapes with corridors. Overall, our findings show strong spatial and temporal discrepancies in population size and trends between network- and landscape-based designs. They suggest that network-based designs may misestimate connectivity benefits compared to real landscapes (Fletcher et al. 2011).

These discrepancies observed in demographic and movement rates between network- and landscape-based treatments reinforce the idea that understanding the processes involved in population responses to habitat fragmentation is essential for the proper design of conservation-targeted landscapes. Although network experiments offer valuable theoretical insights, their applied significance may be limited as they oversimplify fragmented landscapes and disregard the landscape matrix as a fundamental aspect of connectivity conservation (Baum et al. 2004). Indeed, the mortality and permeability of the landscape matrix are elements of landscape connectivity that have long been neglected but are now gaining in importance (Fletcher et al. 2024; Argote et al. 2025). As it is now recognized that real fragmented landscapes are rarely well described by the patch-corridor conceptual model, but require more nuanced approaches (Brudvig 2017; Haddad et al. 2017), corridors embedded in a highly-resistant matrix should not be considered channels and cannot be modeled as such (Valente et al. 2023).

Overall, while our results confirm the potential benefits of corridors for populations at the landscape scale, particularly in terms of movement and spatial distribution, they also highlight that these benefits are strongly limited by the landscape context (distance between patches and matrix resistance). A better understanding of the movement and demographic processes can help to delineate landscapes of conservation interest more effectively. The novel type of miniature landscape-scale experiment presented here enables us to explore the effects of habitat quantity and spatial organization, and matrix resistance simultaneously. This provides more accurate theoretical insights to meet the current needs of spatially explicit conservation biology.

## Supplementary Information

Below is the link to the electronic supplementary material.Supplementary file1 (DOCX 1756 KB)Supplementary file2 (XLSX 1238 KB)

## Data Availability

The data supporting the findings of this study are available upon request by contacting the authors directly.
