# Supplementary material for: Beyond network-based approaches: uncovering the true cost of connectivity
Source: Landsc Ecol. 2026 May 28;41(8):136. doi: 10.1007/s10980-026-02382-3 (PMC13437652; doi:10.1007/s10980-026-02382-3)
Supplement: Supplementary file 1 [file 10980_2026_2382_MOESM1_ESM.docx]

**Supplementary Information** **for:**

article title: Beyond network-based approaches, uncovering the true cost of connectivity

journal name: Landscape Ecology

author names: Karolina Argote, Benoît Geslin, Mathieu Santonja, Lucyle Hernandez-Possémé, Mailys Queru, Cécile H. Albert

affiliation : Aix Marseille Univ, CNRS, IRD, Univ Avignon, IMBE, Marseille

e-mail address of the corresponding author: [cecile.albert@imbe.fr](mailto:cecile.albert@imbe.fr)

**Table of Contents:**

| **SM-1. Selection of a gradient of matrix resistance** | Page 2 |
| --- | --- |
| **SM -2. YOLO-Collembola, an AI-based tool trained to detect *F. candida*** | Page 5 |
| **SM -3. Estimation of body length for *F. candida* and size threshold** | Page 8 |

**SM-1. Selection of a gradient of matrix resistance**

**Rationale**

Matrix resistance influences how individuals move between habitat patches. Establishing a resistance gradient is crucial to simulate different matrix conditions and study how matrix characteristics impact dispersal and colonization success, which is key for understanding population responses to landscape fragmentation (Ricketts, 2001).

**Objectives**

To establish a gradient of matrix resistance, we assessed how different materials affected the ability of Folsomia candida to reach habitat patches when released on them.

**Materials and methods**

We constructed small artificial landscapes measuring 25 × 25 cm using wooden squares. These landscapes were covered with different materials to simulate different types of matrices. The habitat patches consisted of pucks made of a mixture of plaster of Paris and activated charcoal at a ratio of 9:1 (the same medium used for *F. candida* rearing in the laboratory). Each puck had a diameter of 3.4 cm and was topped with a disc of decomposing oak litter that had been pre-saturated for 24 h in a dilution of 200 ml of water with 2 g of yeast granules. The pucks were saturated with water prior to each run (Argote et al. 2025)

Each landscape contained four habitat patches, spaced 13 cm apart. Individuals were released in the center of these landscapes, corresponding to a distance of 4.8 cm from each one of the pucks. Previous research had demonstrated that *F. candida* could easily travel this distance within a few minutes on a suitable matrix (Argote et al. 2023).

The boundaries of the landscapes were materialized by a line of silicone, which was chosen for its non-lethal nature to Collembola. This barrier was implemented to prevent individuals from escaping the landscapes. During the experiment, the landscapes were placed in a covered container with a transparent lid to prevent dehydration of the carbon pucks and to allow for monitoring.

In each landscape, 20 individuals were released. The release was facilitated by a hollow tube in which the individuals were acclimated for a 2-min period before tube was removed and observations began. Observations were made at 10, 30, 60, and 120 minutes intervals. We recorded the number of alive and dead individuals, as well as their respective locations (whether in the matrix or within a specific habitat patch). We tested 34 different materials, replicated 3 times, for a total of 102 runs (Table S1.1). We then compared materials based on the average success rate in reaching habitat areas after release.

**Results and interpretation**

As a result, we observed a gradient of matrix resistance according to the different materials tested (Table S1.1). Organic cotton fabric and wood allowed almost all individuals (>80%) to successfully reach a habitat patch. Aluminum foil and plastic garbage bags prevented any individual from reaching a suitable habitat patch. Variability existed among the three runs for each material type, but the gradient remained largely consistent across the runs. A linear model assessed the effect of matrix type on colonization success. The analysis revealed a statistically significant difference in mean colonization success between materials (F-statistic = 38.89, P <0.0001, R^2^ = 0.81, Fig. S1.2).

*Table S1.1. List of tested materials ranked by increasing success rate*

| **n** | **Material** | **Material abbreviation** | **Mean success rate** |
| --- | --- | --- | --- |
| 1 | Organic Cotton Fabric | fabr | 0.925 |
| 2 | Triplex wood Pine 3mm | wood | 0.821 |
| 3 | Baking Paper | bake | 0.733 |
| 4 | Silver Metal Tin Box Lids | meta | 0.688 |
| 5 | Biodegradable Cling Wrap 0.1mm | film | 0.596 |
| 6 | Watercolor Paper (100% Cellulose 218gsm) | pap2 | 0.554 |
| 7 | Watercolor Paper (100% Cotton Cold Pressed 300gsm) | pap1 | 0.533 |
| 8 | Beeswax food wrap organic cotton | waxe | 0.488 |
| 9 | Cast Acrylic 3mm sheet | acry | 0.479 |
| 10 | Kraft Paper 0.5mm (packaging type) | kraf | 0.438 |
| 11 | Tempered Glass 5mm | glas | 0.429 |
| 12 | Cork sheet 3mm | cork | 0.379 |
| 13 | Plastic Bubble Wrap | bubl | 0.288 |
| 14 | Acetate sheet 0.2mm | ptbl | 0.288 |
| 15 | Ripstop Nylon Kite Fabric | kite | 0.275 |
| 16 | Cardboard Sheet 200 g.sm-1 | car1 | 0.258 |
| 17 | Foam sheet 2mm | mou | 0.238 |
| 18 | Elastic Cotton Fabric | elas | 0.225 |
| 19 | MDF-Medium Density Fiberboard 6mm | mdff | 0.221 |
| 20 | Corrugated Cardboard Sheet 2mm | car2 | 0.204 |
| 21 | Industrial Oilcloth | oilc | 0.200 |
| 22 | Cardboard Sheet 100 g.sm-1 | cart | 0.158 |
| 23 | Fleece fabric | pola | 0.154 |
| 24 | Crepe paper | crep | 0.154 |
| 25 | Bridal Satin Fabric | sati | 0.150 |
| 26 | Thick Cotton Fabric | thic | 0.138 |
| 27 | Sandpaper 40 grit | san1 | 0.125 |
| 28 | Silicone baking sheet | sili | 0.117 |
| 29 | Felt fabric 2mm | felt | 0.100 |
| 30 | Organic Cotton Jersey | jers | 0.083 |
| 31 | Polyethylene garbage | plas | 0.000 |
| 32 | Sandpaper 120 grit | san2 | 0.000 |
| 33 | Polystyrene sheet 5mm | poly | 0.000 |
| 34 | Aluminum foil paper | alum | 0.000 |

Finally, we selected two materials with opposite resistance levels: felt (mean success rate = 10%), a high-resistance material, and organic cotton fabric (mean success rate = 92.5%), a low-resistance material, to construct the landscapes for the experiment presented in the main text. Corridors and stepping stones were made of organic cotton fabric, while the high-resistance matrix was represented by felt.

Fig. S1.2. Success rate in patch colonization according to material types. Points represent mean success rates and bars display the standard deviations (N=3). Materials with a high success rate in patch colonization are shown in green, with an intermediate success rate in patch colonization in orange, and with a low success in patch colonization in red.

**SM -2. YOLO-Collembola, an AI-based tool trained to detect *F. candida***

**Rational**

AI and deep learning techniques have shown great potential for ecological applications, offering improved accuracy and adaptability (Christin et al., 2019). In particular, convolutional neural networks (CNNs)—such as YOLO, R-CNN, SSD, and YOLT—can learn to recognize image features when properly trained, enabling accurate and efficient organism detection and counting (Weinstein, 2018). Among these models, YOLO stands out as the fastest and most suitable for real-time analysis under dynamic laboratory conditions, as it recognizes objects based on their shape and features.

**Objective**

To develop a workflow using a modified version of YOLOv5 (GitHub - ultralytics/yolov5) for detecting and counting living organisms in laboratory experiments involving soil arthropods. The workflow consists of dataset annotation, model training, and evaluation.

**Materials and methods**

We developed a replicable workflow for training YOLOv5 to detect and count *Folsomia candida* individuals from images taken during laboratory experiments (Argote, Santonja, Geslin, Miranda-Fuentes, & Albert, in preparation):

i. **Image acquisition:** We selected 200 high-resolution images from microcosm experiments. These microcosms consisted of cylindrical plastic vials (5.5 cm diameter × 9.5 cm height) with a 3-cm deep flat black mixture of plaster of Paris and activated charcoal (9:1 ratio). Images were captured at a resolution of 6048×4024 pixels using a Nikon NIKKOR Z 24-70mm f/2.8 S digital camera, positioned consistently under uniform lighting conditions provided by 16W, 650 lumen LED bulbs.
ii. **Manual labeling:** *Folsomia candida* individuals were annotated using LabelMe software. This process involved drawing individual bounding boxes around each observed F. candida and assigning a label to each rectangle to identify it as *Folsomia candida.*
iii. **Dataset preparation:** Images were resized to 640×640 and 1280×1280 pixels and we divided our initial dataset (N = 200) into 60% for training, 15% for validation, and 25% for the testing (percentages based in previous studies Zhou, 2022).
iv. **Data augmentation:** We applied augmentation techniques (flipping and rotating) to increase the training set from 120 to 337 images, while keeping the validation and test sets constant.
v. **Model training**: We training the model YOLOv5 *m* variant with 1280×1280 pixels image resolution.
vi. **Model evaluation:** We assessed the performance of the model on the test dataset (N = 50 unseen images). Automatic counts were compared with manual counts through regression analysis, evaluating the coefficient of determination (R²), which measures the strength of the correlation between the automatic and manual counts, indicating how well the model's predictions align with the ground truth. Additionally, we calculated the precision (P) based on the number of true positives (correct detections of Folsomia candida) and false positives (instances where the model incorrectly identified an object as Folsomia candida) using the formula:

Precision (P)$=\frac{True positives}{True positives + False Positives}$

This automated approach demonstrated high accuracy in detecting *F. candida* and can be used for efficient population monitoring in laboratory settings. The calibrated model was then used to count individuals in the experiments presented in the main text of this research article.

All analyses and computations were performed in Python 3.8.6 (Python Software Foundation, 2023) using the libraries: NumPy (Harris et al., 2020) and Pandas (McKinney, 2010) for data handling and preprocessing, Matplotlib (Hunter, 2007) and Seaborn (Waskom, 2021) for generating graphs, and Scikit-learn (Pedregosa et al., 2011) for statistical analysis and metrics computation. OpenCV (Bradski, 2000) was used for image processing and visualization, while PyTorch (Paszke et al., 2019) served as the core framework for deep learning tasks.

**Results and interpretation**

The linear regression analysis comparing the YOLOv5-l-1280px automatic counts with manual counts using the test dataset (*N = 50*), showed a strong correlation, with a 𝑅^2^ value of 0.97, a slope of 1.03096 ± 0.026151 and an intercept close to **0** (0.06341±2.03448). (Fig. S2.1).

Figure S2.1. Correlation between manual and YOLOv5-l-1280px automatic counts on a log scale.

While manual labelling identified 2009 individuals in the test set, the model detected only 1846 of them. A detailed curation of the results revealed that 88% of the detections corresponded to true positives, 4% to false positives, and 12% to omissions (i.e., false negatives, when the model failed to detect present individuals). However, detection accurancy varied among images, with large discrepancies observed in photos containing fewer individuals (Fig. S2.2).

This test dataset was randomly selected from the training dataset but was not seen by YOLOv5 prior to analysis. Within this dataset, 33 images contained individuals and were manually curated, while 17 images with no individuals were correctly classified by YOLOv5-l-1280px

Figure S2.2. Data curation of automatic detections on the test set. True positives are shown in blue, false positives in green, and omissions in red.

Overall, the YOLOv5-l-1280px model provided an accurate estimate of the absolute number of F. candida in each image, achieving 90.4% true positive detections. However, manual curation identified 6.2% omissions (individuals that were not detected) and 6.2% false positives (misclassified objects). Omissions were primarily due to individuals being obscured by overlapping bounding boxes, while false positives resulted from the misclassification of other objects as *F. candida*.

**SM -3. Estimation of body length for *F. candida* and size threshold**

In order to identify adults and juveniles in the experiment we followed these steps:

1 – Identify individuals in habitat patches with Yolov5 (Fig. S3.1) – this leads to each individuals being tagged with a rectangle that encompasses its body (see photo below).

Figure S3.1. Identification of individuals with YOLO-Collembola based on weekly pictures of habitat patches. Identified individuals are surrounded with a red box.

2 – Collect rectangle dimensions, and estimate its diagonal length in pixels, then convert in mm with a predefined scale (not-shown).

3 – Create histograms by weeks (Fig. S3.2). The smaller values obtained at week 1 were double checked to remove false positive (few false positive can emerge due to small particles and debris).

Figure S3.2. Weekly histograms of body-length. The vertical line indicates the threshold of 0.85 mm and the pink area an error interval of +/-0.05 mm around the threshold.

4 – Healthy adults at week 1 measure on average 1.36 mm, most being between 1.02 and 1.65 mm (10 and 90% quantiles), the smallest observed reached 0.9 mm.

5 – In the subsequent weeks, body length distributions are bimodal with the emergence of juveniles. There is a clear threshold of size between 0.8 and 0.9 mm. We thus selected 0.85 as a threshold to distinguish between juveniles or sub adults and healthy, well-developed adults.

**References**

Argote, K., Albert, C. H., Geslin, B., Biryol, C., & Santonja, M. (2023). Effects of litter quality on foraging behaviour and demographic parameters in *Folsomia candida* (Collembola). Ecology and Evolution, 13, e10420. <https://doi.org/10.1002/ece3.10420>.

Argote, K., Geslin, B., Santonja, M., & Albert, C. H. (2025). The amount of reachable habitat determines populations fate. Ecography. Advance online publication. <https://doi.org/10.1002/ecog.08080>.

Argote, K., Santonja, M., Geslin, B., Miranda-Fuentes, J., & Albert, C. H. (in preparation). Real-time soil mesofauna monitoring with YOLOv5: From population dynamics to ecosystem functioning. Manuscript in preparation for Ecological Informatics.

Bradski, G. (2000). The OpenCV Library. Dr. Dobb's Journal of Software Tools.

Christin, S., Hervet, É., & Lecomte, N. (2019). Applications for deep learning in ecology. Methods in Ecology and Evolution, 10(10), 1632–1644. <https://doi.org/10.1111/2041-210X.13256>.

Harris, C. R., Millman, K. J., van der Walt, S. J., Gommers, R., Virtanen, P., Cournapeau, D., ... & Oliphant, T. E. (2020). Array programming with NumPy. Nature, 585(7825), 357–362. <https://doi.org/10.1038/s41586-020-2649-2>.

Hunter, J. D. (2007). Matplotlib: A 2D graphics environment. Computing in Science & Engineering, 9(3), 90–95. <https://doi.org/10.1109/MCSE.2007.55>.

McKinney, W. (2010). Data structures for statistical computing in Python. In Proceedings of the 9th Python in Science Conference (Vol. 445, pp. 51–56).

Paszke, A., Gross, S., Massa, F., Lerer, A., Bradbury, J., Chanan, G., ... & Chintala, S. (2019). PyTorch: An imperative style, high-performance deep learning library. In Advances in Neural Information Processing Systems, 32.

Pedregosa, F., Varoquaux, G., Gramfort, A., Michel, V., Thirion, B., Grisel, O., … & Duchesnay, É. (2011). Scikit-learn: Machine learning in Python. Journal of Machine Learning Research, 12, 2825–2830.

Python Software Foundation. (2023). Python (Version 3.8.6). <https://www.python.org/>

Ricketts, T. H. (2001). The matrix matters: Effective isolation in fragmented landscapes. The American Naturalist, 158(1), 87–99. <https://doi.org/10.1086/320863>.

Waskom, M. L. (2021). seaborn: statistical data visualization. Journal of Open Source Software, 6(60), 3021. <https://doi.org/10.21105/joss.03021>.

Weinstein, B. G. (2018). A computer vision for animal ecology. Journal of Animal Ecology, 87(3), 533–545. <https://doi.org/10.1111/1365-2656.12780>.

Zhou, Z. (2022). Detection and counting method of pigs based on YOLOV5_Plus: A combination of YOLOV5 and attention mechanism. Mathematical Problems in Engineering, 2022, Article 7078670. <https://doi.org/10.1155/2022/7078670>.
